# Discoid Lupus Erythematosus: A Cross-Sectional Study From the Sindh Institute of Skin Diseases, Karachi, Pakistan

**DOI:** 10.7759/cureus.11201

**Published:** 2020-10-27

**Authors:** Erum Ashraf, Afza N Ghouse, Sitwat Siddiqui, Sana Siddiqui, Zara Khan

**Affiliations:** 1 Dermatology, Institute of Skin Diseases Sindh, Karachi, PAK; 2 Dermatology, Patel Hospital, Karachi, PAK; 3 Dermatology, Dr. Ziauddin Hospital, Karachi, PAK; 4 Dermatology, Memon Medical Institute Hospital, Karachi, PAK; 5 Dermatology, Medical Glamor Clinics, Riyadh, SAU

**Keywords:** lupus profundus, tumid lupus, chilblain lupus, lichen planus, discoid lupus erythematosus (dle)

## Abstract

Introduction

Discoid lupus erythematosus (DLE) is the most common form of cutaneous lupus erythematosus. It is a chronic, scar-forming, photosensitive autoimmune dermatosis presenting with erythematous and scaly lesions. Predisposed areas include sun-exposed areas like the nose, forehead, and cheeks, as well as the upper body and extremities. The histological findings are typical, with interface dermatitis. Immunoglobulin M (IgM) and immunoglobulin G (IgG) are the most common deposits in the dermoepidermal junction of the involved skin. The most common treatments used are sunscreens, topical corticosteroids, and antimalarials. Immunosuppressive agents, thalidomide, dapsone, and retinoids can be used in refractory cases. The aim of this study was to study the clinicopathologic patterns of DLE in patients presenting to the Institute of Skin Diseases in Sindh, Karachi.

Methods

A total of 53 consecutive patients with DLE meeting the inclusion criteria were evaluated between February 18, 2018 to March 2, 2019 at the Institute of Skin Diseases. Patients with clinical suspicion of DLE were evaluated and studied prospectively after written informed consent was obtained. Information was then collected from their medical histories, physical examination records, and laboratory investigation reports.

Results

A total of 53 consecutive patients with clinical and/or histological diagnosis of DLE was included in this study, out of which 75.5% (40) were females with a male to female ratio of 1:3.1. The mean age of the patients at the time of presentation was 36.02 ± 10.04 years, ranging from 14 to 65 years. More than half of the patients (35, 66.0%) were under 40 years of age and 20.8% (11) had a positive family history of DLE. DLE was localized in 36 patients (67.9%) and exposure to the ultraviolet radiation (UVR) was found to be the most frequent induced factor in 46 patients (86.8%), followed by stress which was observed in 14 patients (26.4%). The distribution of commonly affected sites were the face (81.1%), the limbs (71.7%), and the scalp (48.4%) of the patients. Serology antinuclear antibody (ANA) was positive in 56.6% and serology anti-double-stranded deoxyribonucleic acid antibodies (anti-dsDNA) were positive in 45.3% of patients. Smoking, as an induced factor, was more commonly observed among male patients as compared to the female patients with a proportion of 53.8% vs. 2.5%, p < 0.001, while stress was more common among female patients with a proportion of 35% vs. 0%, p = 0.013, respectively. Histopathology with direct immunofluorescence was done in 33 cases which included cases with negative serology or where the diagnosis was in doubt clinically. The main histopathological features observed were periadnexal and perivascular dermal infiltrates, basal cells vacuolization, epidermal atrophy, hyperkeratosis, and follicular plugging. The commonest morphological form observed was the classic discoid plaque form.

Conclusion

Clinical patterns of DLE in our population comprises of female dominance. Exposure to UVR was the leading inducing factor. The face and limbs were the most commonly involved sites, and the majority of the patients had localized DLE with positive ANA in more than half of those patients. The importance of limiting ultraviolet radiation exposure and toxins (drugs and smoking) should be emphasized in our population.

## Introduction

Discoid lupus erythematosus (DLE) is a benign inflammatory disorder that is autoimmune in origin. The disease involves mainly the sun-exposed areas of the body, especially the face and scalp, with variable clinical presentations and is characterized by scaly, well-defined red plaques healing with scarring and pigmentary changes [1]. It is approximately three times more common in females as compared to males and most of these females are 40 - 50 years of age [2]. The disease is broadly categorized into a localized variant involving mainly the face and scalp areas and a disseminated form which is widespread extending below the neck area [3]. Other variants include chilblain lupus which is characterized by red to violaceous lesions on the acral parts of the body induced by cold, leading to ulceration and scarring, and lupus profundus characterized by painful subcutaneous nodules and plaques followed by atrophy of fat [4-5]. Other less common forms are hypertrophic or verrucous forms, discoid lupus erythematosus, and lichen planus overlap form, tumid form, linear form, etc. The diagnosis of DLE is usually made clinically. For confirmation of diagnosis, serological and histopathological analysis with immunofluorescence are done. Common serological tests include an antinuclear antibody test (ANA) which is positive in 20% to 30% cases (when tested on human cell lines) and anti-double-stranded DNA antibodies (anti-dsDNA) which is positive in 5% to 20% cases. Other serological tests, including precipitating anti-Sjögren's-syndrome-related antigen A (Ro/SSA) and anti-Sjögren's-syndrome-related antigen B (La/SSB) autoantibodies, are rare in DLE [6]. However, low levels of anti-Ro/SSA antibodies detected by enzyme-linked immunoassays are more common [6]. The histopathologic features of DLE include epidermal atrophy, hyperkeratosis, follicular plugging, vacuolar degeneration of basal cells, thickening of the basement membrane, perivascular, periappendiceal lymphocytic cell infiltrate, and sometimes mucin deposition [7]. For further confirmation, we looked for the deposition of immunoreactants by direct immunofluorescence at the dermoepidermal junction. These immunoreactants, which include immunoglobulins with or without complements, are usually positive in the granular pattern at the dermoepidermal junction of lesional skin in around 90% of cases [8-9].

There are scanty studies on DLE in the Pakistani population which is said to be the commonest variant of cutaneous lupus erythematosus worldwide. Knowledge of its clinical spectrum, epidemiology, and diagnostic tests are important for a timely diagnosis and hence early treatment of the disease since a delay in diagnosis is associated with complications and poor emotional impact. 

## Materials and methods

This was a cross-sectional study with a total of 53 consecutive patients of both genders, aged between 14 to 65 years, who were admitted with clinical suspicion of discoid lupus erythematosus in both inpatient and outpatient units of the Institute of Skin Diseases, Sindh, Karachi. The sample size was calculated using the World Health Organization (WHO) software, version 2.0. The study by Bajaj et al. was taken as a reference study for sample size calculation, in which the face was found to be the commonest site, accounting for 54.5% [10]. As our sample size was small, we had to set the margin of error by 14% rather than the standard 10% and confidence interval as 94% to calculate the sample size. The sample size for our study came out to be 47 patients which, after adjusting 10% as lost to follow-up patients, a final total of 53 patients were recruited during the study period. These patients were evaluated between February 18, 2018 to March 2, 2019 at the Institute of Skin Diseases and studied prospectively. Patients with systemic manifestations of the disease and mixed connective tissue disorders were excluded from the study. Those patients who were younger than 14 and older than 65 years of age, pregnant patients, and those not willing to give consent were also excluded. Written informed consent was obtained from all patients.

The medical records of all patients were obtained in detail according to the proforma which included age, gender, family history, distribution of the lesions of DLE, and type of DLE. Induced factors like ultraviolet light, viral infections, drugs, smoking, and trauma were also recorded. The distinction between localized and disseminated lesions were solely made on cutaneous involvement; lesions limited to the head and neck were labeled as the localized variety and lesions beyond the neck were labeled as the disseminated variety. The routine laboratory serology blood tests (ANA, dsDNA, anti-Ro SSA, anti-La SSB, and anti-Sm) were analyzed by standard methods. Serum ANA was tested preferably by human epithelial type 2 (HEp-2) cells using immunofluorescence technique or by enzyme-linked immunosorbent assay (ELISA) method. Anti-dsDNA antibody levels were analyzed by radioimmunoassay (RIA) or ELISA. Wherever needed, histopathology analysis was done and recorded on the proforma as suggestive or not suggestive of DLE. Tissues for histological examination were fixed in 10% buffered formalin and processed for hematoxylin and eosin stain. Histopathology findings were further confirmed on the detection of immunoreactants at the dermoepidermal junction by the direct immunofluorescence technique in which the biopsy samples were placed in Michel's medium and stored at 4°C until further processed. In the statistical analysis, the descriptive variables were presented as frequency, percentage, mean or standard deviation, and the results were reported. For comparison of gender and groups among the different characteristics of the patients, the chi-squared test/Fisher exact test were analyzed by using the Statistical Package of Social Sciences (SPSS), version 21 (IBM SPSS Statistics, Armonk, NY).

## Results

A total of 53 consecutive patients with DLE was included in this study, out of which 75.5% (n = 40) were females with a male to female ratio of 1:3.1. The mean age of the patients at the time of presentation was 36.02 ± 10.04 years (ranging from 14 to 65 years). More than half of the patients (n = 35, 66.0%) were under 40 years of age and 11 (20.8%) had a positive family history. DLE was localized in 67.9% (n = 36) of the patients and exposure to the ultraviolet radiation (UVR) was found to be the most frequent induced factor, 86.8% (n = 46), followed by stress which was observed in 26.4% (n = 14) of the patients. Among multiple response categories in the face, the cheek was found to be the commonest site involved in 76.7% (n = 33) cases, followed by the nose 60.5%, forehead 48.8%, eyelid 39.5%, and trunk 37.2% cases, respectively.

The distribution of commonly affected sites are the face in 81.1%, limbs in 71.7%, and scalp in 48.4% of the patients. Serology antinuclear antibody (ANA) was positive in 56.6% and serology anti-dsDNA was positive in 45.3%. Clinical patterns of discoid lupus erythematosus by gender are presented in Table 1.

**Table 1:** Clinical Patterns of Discoid Lupus Erythematosus by Gender * Significant at 5% ^1  ^Viral infections (herpes zoster virus, old smallpox vaccination) ^2 ^Drugs (isoniazid, penicillamine. griseofulvin, dapsone, hydralazine) ANA: antinuclear antibody; DNA: deoxyribonucleic acid

Characteristics	Total	Gender		P-value
		Male	Female	
Sample size (N)	53	13	40	-
INDUCED FACTORS				
Ultraviolet radiation (UVR)	86.8% (46)	76.9% (10)	90% (36)	0.226
Viral infection^1^	13.2% (7)	7.7% (1)	15% (6)	0.499
Drugs^2^	9.4% (5)	0% (0)	12.5% (5)	0.18
Smoking	15.1% (8)	53.8% (7)	2.5% (1)	< 0.001*
Trauma	9.4% (5)	7.7% (1)	10% (4)	0.805
Stress	26.4% (14)	0% (0)	35% (14)	0.013*
TYPES				
Localized	67.9% (36)	38.5% (5)	77.5% (31)	0.009*
Disseminated	32.1% (17)	61.5% (8)	22.5% (9)	
SITE OF INVOLVEMENT				
Scalp	48.4% (16)	50% (3)	48.1% (13)	0.52
Face	81.1% (43)	92.3% (12)	77.5% (31)	0.236
Cheeks	76.7% (33)	83.3% (10)	74.2% (23)	0.209
Nose	60.5% (26)	66.7% (8)	58.1% (18)	0.300
Forehead	48.8% (21)	41.7% (5)	51.6% (16)	0.922
Ears	39.5% (17)	33.3% (4)	41.9% (13)	0.908
Trunk	37.2% (16)	33.3% (4)	38.7% (12)	0.958
Limbs	71.7% (38)	69.2% (9)	72.5% (29)	0.82
Upper limbs	57.9% (22)	22.2% (2)	69% (20)	0.013*
Lower limbs	42.1% (16)	77.8% (7)	31% (9)	
SEROLOGY ANA				
Positive	56.6% (30)	30.8% (4)	65% (26)	0.031*
Negative	43.4% (23)	69.2% (9)	35% (14)	
SEROLOGY DNA				
Positive	45.3% (24)	38.5% (5)	47.5% (19)	0.57
Negative	54.7% (29)	61.5% (8)	52.5% (21)	

Smoking as an induced factor was more commonly observed among male patients as compared to the female patients with a proportion of 53.8% versus 2.5% (p < 0.001), while stress was more common among female patients with a proportion of 35% vs. 0% (p = 0.013). DLE was localized in more female patients than males with rates of 77.5% vs. 38.5% (p = 0.009); similarly, positive serology ANA was found to be associated with female gender with rates of 65% vs. 30.8% (p = 0.031). However, no major differences in the clinical patterns of DLE were observed for the different age groups of the patients. The clinical patterns of discoid lupus erythematosus by age are presented in Table 2.

**Table 2:** Clinical Patterns of Discoid Lupus Erythematosus by Age * Significant at 5% ^1 ^Viral infection (herpes zoster, old smallpox vaccination) ^2 ^Drugs (isoniazid, penicillamine, griseofulvin, dapsone, hydralazine) ANA: antinuclear antibody; DNA: deoxyribonucleic acid

Characteristics	Age Group (% / n) 14 to 40 years	Age Group (% / n) 41 to 65 years	P-value
Sample size (N)	35	18	-
INDUCED FACTORS			
Ultraviolet radiation (UVR)	88.6% (31)	83.3% (15)	0.594
Viral infection^1^	11.4% (4)	16.7% (3)	0.594
Drugs^2^	8.6% (3)	11.1% (2)	0.765
Smoking	11.4% (4)	22.2% (4)	0.299
Trauma	5.7% (2)	16.7% (3)	0.196
Stress	31.4% (11)	16.7% (3)	0.248
TYPES			
Localized	74.3% (26)	55.6% (10)	0.167
Disseminated	25.7% (9)	44.4% (8)	
SITE OF INVOLVEMENT			
Scalp	42.8% (9)	58.3% (7)	0.322
Face	82.9% (29)	77.8% (14)	0.654
Cheeks	72.4% (21)	85.7% (12)	0.635
Nose	55.2% (16)	71.4% (10)	0.497
Forehead	41.4% (12)	64.3% (9)	0.268
Ears	27.6% (8)	64.3% (9)	0.045*
Trunk	37.9% (11)	35.7% (5)	0.784
Limbs	74.3% (26)	66.7% (12)	0.560
Upper limbs	53.8% (14)	66.7% (8)	0.457
Lower limbs	46.2% (12)	33.3% (4)	
SEROLOGY ANA			
Positive	60% (21)	50% (9)	0.487
Negative	40% (14)	50% (9)	
SEROLOGY DNA			
Positive	45.7% (16)	44.4% (8)	0.93
Negative	54.3% (19)	55.6% (10)	

Histopathology with direct immunofluorescence was done in 33 cases where the diagnosis was in doubt. The main histopathological features observed were periadnexal and perivascular dermal infiltrates (32/33), basal cells vacuolization (30/33), epidermal atrophy (29/33), hyperkeratosis (29/33), and follicular plugging in 27/33 cases. 

Regarding the morphological variants, a classic discoid plaque was present in 36/53 (68%) patients, followed by the hypertrophic form in 7/53 (13%). Discoid lupus erythematosus with lichen planus overlap and lupus profundus forms each were present in 4/53 (7.54%) patients. Linear DLE was diagnosed in 2/53 (4.3%) cases.

## Discussion

Discoid lupus erythematosus (DLE) is the most common type of chronic cutaneous lupus erythematosus. It can present with different clinical patterns. Hence, in this study, we sought to present clinicopathologic patterns of DLE in the Pakistani population [10]. A higher female to male ratio of 3.1:1 among DLE patients is a common finding in this population. Female patients outnumbered male patients not only in our population but it is also a common observation for various other geographies. The most common age group affected was 14 - 40 years of age with a mean age of onset at 29.4 years which is comparable to other studies [10-13]

Along with the various factors leading to DLE, such as exposure to ultraviolet radiation (UVR), toxins (drugs and smoking), and genetic predisposition, it is believed that hormonal factors, such as estrogen, play a causative role and increases the risk of DLE among females [14]. Stress was also a predominant factor associated with disease activity in 80% of cases, an important factor which was also proved by a Western study by Chen et al. [15]. Smoking also showed a strong association with DLE in our study, a factor already proved important as an inducing factor by other studies [16]. Six of our patients developed DLE after intake of anti-tuberculous treatment which is a known drug to induce DLE as proved by other studies, while one patient developed the disease after the intake of hydralazine [17-18].

Occupational or non-occupational exposure to UVR is the most common induced factor of DLE in our population, followed by stress, smoking, and viral infection. These observations were in concordance with the literature [12, 19-20]. A major portion of our population experiences an average longer duration of exposure to UVR due to their affiliation with outdoor occupations, such as labor, farming, small street businesses, and working for daily wages [10]. The release of pro-inflammatory cytokines during the apoptosis of keratinocytes due to UV light induces inflammatory changes resulting in DLE [19]. Along with other environmental factors, smoking is considered to play a significant role in the pathogenesis of autoimmune diseases. It was also found to be associated with DLE development [13, 21].

DLE was observed to be localized in the majority of the patients which was in concordance with the results observed in the past studies [10-13]. Disseminated DLE was more common in male patients as compared to females. Post-inflammatory pigmentation is frequently seen in skin types III to V, which are the commonest skin types in our population, contributing to residual pigmentation of lesions. In our study, hydroxychloroquine and azathioprine drugs were commonly used for the treatment of DLE which was also reported by Wahie et al. [13]. ANA serology was positive in a significant number of patients (56.6%) and was preferably requested on HEp-2 cells to decrease the chances of false-negative results. These results were in contrast to the observations of Bajaj et al. [10] who reported only 17.3% cases with positive ANA serology. A similar study by Gopalan et al. reported 73% of cases with positive ANA serology and a strong association with the female gender [12].

Patients with autoantibodies to ds-DNA and anti-Ro were thoroughly checked for SLE; fortunately, none of these patients had clinical and laboratory evidence of SLE. We compared our results with a large multicentre Western study conducted by Biazar et al. [22], a local study conducted by Bajaj et al. [10], and a literature review done in Saudia Arabia by AlSaif [23] and observed that our results were comparable.

The most common site of lesion was the face, which was also observed in 81.1% of patients in our study. The cheeks, nose, and forehead were the common lesional sites on the face [10, 12]. Limb involvement was surprisingly high in our study (71.7%). The scalp was found to be involved in 37.7% of the patients which was in concordance with the range of frequency, 23.6% to 41%, reported in past studies [24-25].

Histopathology with direct immunofluorescence was done in 33 cases which included cases with negative serology or where the diagnosis was in doubt clinically. The main histopathological features observed were periadnexal and perivascular dermal infiltrates 32/33, basal cell vacuolization 30/33, epidermal atrophy 29/33, hyperkeratosis 29/33, and follicular plugging in 27/33 cases. These are common findings observed in the histopathological examination as mentioned in the literature and are important clues to confirm the diagnosis [25-26].

Regarding the morphological variants, the classic discoid plaque was present in 36/53 (68%), followed by the hypertrophic form in 7/53 (13%) of patients. Discoid lupus erythematosus with lichen planus overlap form and lupus profundus forms each were present in 4/53 (7.54%) of patients. Linear DLE was diagnosed in 2/53 (4.3%) cases. It is said to be a unique and one of the rarest forms of DLE with only a few reported cases in the literature [27-28].

DLE is a disease characterized by inflammatory plaques. Currently, very limited data are available on the effectiveness of available treatment options. No medication has been approved specifically for DLE, although many drugs have been used for the management and treatment of DLE [29-30]. A systematic review of clinical trials summarized the available evidence and concluded that fluocinonide creams can be more effective for DLE skin lesions as compared to hydrocortisone [30]. For complete resolution, hydroxychloroquine and azathioprine were found to have equal efficacy. However, acitretin therapy had a more adverse event rate than hydroxychloroquine. Quality randomized control trials are needed to deduce any conclusion with certainty regarding the treatment of DLE [30].

The findings of this study have to be seen in the light of a few limitations. There are three major limitations in this study that could be addressed in future research. The first is that our study is a single centre experience, the second limitation concerns time constraints and a small number of patients, and the third one relates to limited previous studies locally.

## Conclusions

Clinicopathologic patterns of DLE in our population comprises of female dominance, the face and limbs were the most common sites, exposure to the ultraviolet radiation (UVR) was the leading inducing factor, and the majority of the patients had localized DLE with positive ANA. The importance of limiting ultraviolet radiation exposure and the use of toxins (drugs and smoking) should be emphasized in our population. Similarly, patients with disseminated DLE should be regularly followed as these cases carry a risk of transmission into squamous cell carcinoma. Hence, we conclude that it is very important for healthcare providers, especially dermatologists, to have adequate knowledge about the signs and symptoms of DLE and its variants to ensure early detection and treatment.
